# Diabetes Education and Support Tele-Visit Needs Differ in Duration, Content, and Satisfaction in Older Versus Younger Adults

**DOI:** 10.1089/tmr.2022.0007

**Published:** 2022-05-09

**Authors:** Margaret Greenfield, Diana Stuber, Danielle Stegman-Barber, Karen Kemmis, Belinda Matthews, Carly B. Feuerstein-Simon, Prasenjit Saha, Beth Wells, Teresa McArthur, Christopher P. Morley, Ruth S. Weinstock

**Affiliations:** ^1^ Department of Medicine, SUNY Upstate Medical University, Syracuse, New York, USA.; ^2^ Cecelia Health, New York, New York, USA.; ^3^ Department of Public Health and Preventive Medicine, SUNY Upstate Medical University, Syracuse, New York, USA.

**Keywords:** diabetes, diabetes support, older adults, tele-education

## Abstract

**Background:**

Diabetes education and support are critical components of diabetes care. During the COVID-19 pandemic, when telemedicine took the place of in-person visits, remote Certified Diabetes Care and Education Specialist (CDCES) services were offered to address diabetes education and support. Specific needs for older adults, including the time required to provide education and support remotely, have not been previously reported.

**Methods:**

Adults with diabetes (primarily insulin-requiring) were referred to remote CDCESs. Utilization was individualized based on patient needs and preferences. Topics discussed, patient satisfaction, and time spent in each tele-visit were evaluated by diabetes type, age, sex, insurance type, glycosylated hemoglobin (HbA1c), pump, and continuous glucose monitor (CGM) usage. *t*-Tests, one-way analysis of variance, and Pearson correlations were employed as appropriate.

**Results:**

Adults (*n* = 982; mean age 48.4 years, 41.0% age ≥55 years) with type 1 diabetes (*n* = 846) and type 2 diabetes mellitus (*n* = 136, 86.0% insulin-treated), 50.8% female; 19.0% Medicaid, 29.1% Medicare, 48.9% private insurance; mean HbA1c 8.4% (standard deviation 1.9); and 46.6% pump and 64.5% CGM users had 2203 tele-visits with remote CDCESs over 5 months. Of those referred, 272 (21.7%) could not be reached or did not receive education/support. Older age (≥55 years), compared with 36–54 year olds and 18–35 year olds, respectively, was associated with more tele-visits (mean 2.6 vs. 2.2 and 1.8) and more time/tele-visits (mean 20.4 min vs. 16.5 min and 14.8 min; *p* < 0.001) as was coverage with Medicare (mean 2.8 visits) versus private insurance (mean 2.0 visits; *p* < 0.001) and lower participant satisfaction. The total mean time spent with remote CDCESs was 53.1, 37.4, and 26.2 min for participants aged ≥55, 36–54, and 18–35 years, respectively. During remote tele-visits, the most frequently discussed topics per participant were CGM and insulin pump use (73.4% and 49.7%). After adjustment for sex and diabetes type, older age was associated with lack of access to a computer, tablet, smartphone, or internet (*p* < 0.001), and need for more education related to CGM (*p* < 0.001), medications (*p* = 0.015), hypoglycemia (*p* = 0.044), and hyperglycemia (*p* = 0.048).

**Discussion:**

Most remote CDCES tele-visits were successfully completed. Older adults/those with Medicare required more time to fulfill educational needs. Although 85.7% of individual sessions lasted <30 min, which does not meet current Medicare requirements for reimbursement, multiple visits were common with a total time of >50 min for most older participants. This suggests that new reimbursement models are needed. Education/support needs of insulin-treated older adults should be a focus of future studies.

## Introduction

Diabetes self-management education and support (DSMES) provided by qualified health care professionals, including Certified Diabetes Care and Education Specialists (CDCESs), has been shown to improve diabetes self-management and outcomes,^1–14^ but health care coverage is variable and many insurers will only reimburse for education sessions that last for at least 30 min.^13–15^ In addition to lack of access, barriers to participation include distance, cost, and convenience (i.e., taking time off from work).^13–16^

Some barriers to receiving professional care for diabetes can be ameliorated by telehealth.^17^ Telehealth has the potential to reach many more people with diabetes (PWD), and numerous studies have demonstrated efficacy.^7^ Internet access as well as access to computers, tablets, and/or smartphones and technological acumen correlate with age and education level.^18,19^ The specific needs for education and support of older adults with insulin-requiring diabetes using telehealth have not been well studied. The time required to meet diabetes education and support needs of older adults using telemedicine, including needs related to the use of new and remote monitoring devices and insulin delivery systems, is important to understand but has not been previously reported.

With the onset of the COVID-19 pandemic, in-person adult diabetes care visits ceased, and remote provider and CDCES visits were started. The CDCESs provided remote education and support, including device education (insulin pump, continuous glucose monitor [CGM], and meter support), lifestyle, and psychosocial support, and addressed the management of hypoglycemia and hyperglycemia, financial concerns, sick day management, and the insulin injection technique.

Previous studies have shown patient satisfaction with provider telemedicine visits to be high.^20–25^ Patients preferred telemedicine for reasons such as convenience, illness, or wanting to avoid waiting rooms for risk of becoming infected with COVID-19 during the pandemic.^26^ However, there is a relative lack of data specifically pertaining to patient satisfaction with remote diabetes education and support across the lifespan.

In this study, real-world experience working with remote CDCESs to facilitate care for PWD on complex insulin regimens is reported, and differences in the duration and content of these tele-visits, and patient satisfaction are examined in older versus younger adults.

## Materials and Methods

### Research design

A cross-sectional design was used to assess the utilization, impact, and satisfaction related to remote diabetes education and support tele-visits.

### Participants

One diabetes center in Syracuse, New York referred adults (ages ≥18 years) to a team of remote CDCESs to provide diabetes education, training, and support during the early months of the COVID-19 pandemic (from April 2020 to September 2020). Referrals were made for any English-speaking adult with type 1 diabetes (T1D) mellitus with an upcoming medical visit. Based on needs, providers could refer individuals with type 2 diabetes (T2D) mellitus, primarily using multiple daily insulin injections. Adults who were non-English speaking were provided services by the diabetes center using translation services that were previously in place. This study was reviewed by The Institutional Review Board for the Protection of Human Subjects at SUNY Upstate Medical University.

### Diabetes care, education, and support tele-visits

A remote team of CDCESs provided diabetes education, training, and support. This service was free of charge to participants, with the cost being covered by COVID relief funds given by the Leona M. and Harry B. Helmsley Charitable Trust. These funds permitted the provision of unrestricted comprehensive DSMES based on participants' needs and level of desire. Medical care provided by physicians and advanced practice practitioners was billed as usual.

The following information was made available to the remote CDCESs: participants' age, sex, type of diabetes, medications, diabetes devices, most recent glycosylated hemoglobin (HbA1c; all HbA1c values ≥14.0% were recorded as 14.0%), and contact information. Additional information was provided as needed, including any known potential barriers to education, the presence of diabetes complications, and urgency of the referral. This information was uploaded to a secure cloud content management and file sharing service.

The initial contact attempt was made by phone, and follow-up communications between the remote CDCESs and the participant were performed by either phone or video. If the CDCES determined a video tele-visit would be helpful for the participant, this would be offered. The team of remote CDCESs were detailed and used talk-back methods to help the individual use their software and devices as needed. Video visits were used for the majority of individuals who were started on CGM. The CDCESs were also able to instruct individuals using the phone while also referring the participant to additional starter guides and videos as indicated. Up to three attempts were made to reach each participant.

Participants were considered to have a tele-visit with a CDCES when any diabetes education or medical information was reviewed or discussed. When tele-visits were performed by video, the platform Zoom for Healthcare was used. The remote CDCES team's call center platform was used to obtain the duration of each tele-visit (minutes).

Information collected by the CDCESs included the frequency of visits, topics discussed, device data, any dosing changes made within approved parameters, and/or urgent recommendations made by the remote team. This information was uploaded into the EMR daily, and it was forwarded to the participant's provider for review. A CDCES at the diabetes center was always available to receive calls from the remote CDCESs for urgent issues.

After the first 1–2 months, titration of insulin doses by the remote CDCES was initiated within pre-defined parameters. If the remote CDCES had high priority recommendations or concerns after contacting a participant, such as dose change recommendations outside of the set parameters, this information was communicated to the diabetes center CDCES to address. A virtual huddle between the primary site and remote CDCES teams was initially conducted daily, as new workflows and procedures were initiated, and then 1–3 times per week for the subsequent 2–3 months.

### Participant satisfaction with remote CDCES tele-visits

Surveys were completed between May 2020 and August 2020 after at least one visit with a remote diabetes educator. Participants were asked to complete the survey electronically by using Research Electronic Data Capture (REDCap). If there was no response within 1 week or if there was no e-mail address available, the survey was administered by phone. Each participant was called a maximum of two times.

A phone script was used when calling participants to standardize survey administration. Special attention was given to ensure that little feedback was given by research assistants over the phone. Survey questions used a 5-point Likert scale: strongly disagree (1) through strongly agree (5). Participants could also provide additional comments. Questions were developed with guidance from previously used telehealth surveys such as the Telemedicine Satisfaction Questionnaire^27^ and Telehealth Usability Questionnaire.^28^

### Data analyses

Differences between age (in years), number of people in each insurance category (commercial, Medicare, or Medicaid), sex (male or female), and type of diabetes (T1D vs. T2D) were analyzed by using the *t*-test. The mean number of tele-visits by sex and differences in survey item mean scores by age was assessed by *t*-test or one-way analysis of variance, as appropriate. Pearson correlation coefficients were calculated to assess the associations between the number of tele-visits, the duration of all tele-visits for each participant, and average call time duration and age.

To assess the relationship between Likert scaled items and age, Pearson correlation coefficients were used. Bivariate correlation coefficients were calculated for all educational topics (number of times addressed) by age, and partial correlations were also calculated to adjust for sex and diabetes type. Statistical analyses were performed by using SPSS version 27.

## Results

### Participant characteristics

A total of 1301 referrals were made for 1269 adults, 1089 with T1D and 165 with T2D. Excluded from these analyses were those with pre-diabetes (*n* = 7), cystic fibrosis-related diabetes (*n* = 4), and obesity without diabetes (*n* = 4). Of the 1254 participants with T1D and T2D, 982 (78.3%) had at least one visit with a remote CDCES. The others (21.7%) were unable to be reached or declined education services.

Participant characteristics are shown in Table 1. The mean age was 48.4 years (standard deviation [SD] 17.3). Approximately half of the participants were females (50.8%), and commercial insurance was the primary coverage for 48.9% of the participants. The mean HbA1c was 8.4% (SD 1.9; range 4.2–14.0%). There were 64.5% using CGM, 46.6% using insulin pumps, and 18.4% had no access to a computer, tablet, or smartphone.

**Table 1.** Participant Characteristics

	Total participants, *n* (%)
Total participants	982 (100)
Type of diabetes	
T1D	846 (86.2)
T2D	136 (13.8)
Type 2 insulin treated	117 (86.0)
Type 2 non-insulin treated	19 (14.0)
Sex	
Female	499 (50.8)
Insurance	
Commercial	480 (48.9)
Medicare	286 (29.1)
Medicaid	187 (19.0)
Other Gov't	21 (2.1)
Uninsured	8 (0.8)
Ages (years)	
18–35	285 (29.0)
36–54	294 (29.9)
55+	403 (41.0)
Mean age (years), (SD)	48.4 (17.3)
Mean HbA1c % (SD)	8.4 (1.9)
^a^Range	4.2–14.0
Pump use	458 (46.6)
CGM use	633 (64.5)
No access to computer, tablet, smartphone	181 (18.4)

^a^ Fourteen percent includes >14%.

CGM, continuous glucose monitor; HbA1c, glycosylated hemoglobin; SD, standard deviation; T1D, type 1 diabetes; T2D, type 2 diabetes.

### Tele-visit characteristics

**FIG. 1.**
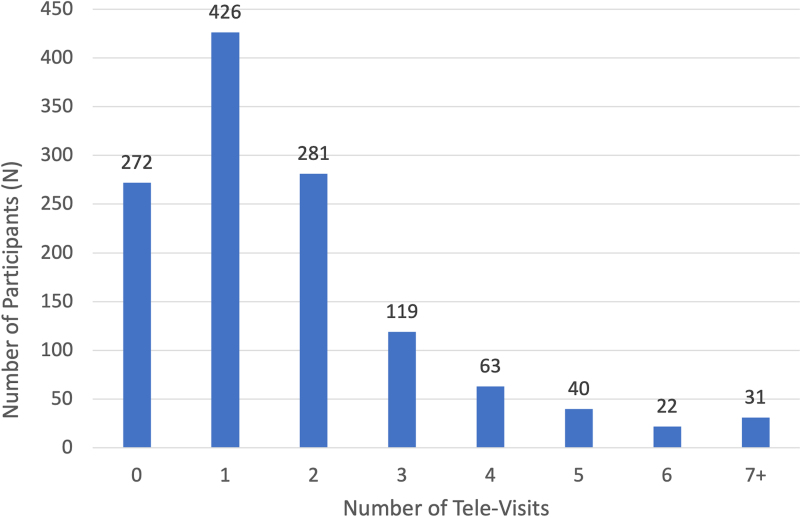
Frequency of diabetes care and education tele-visits.

A total of 2203 tele-visits were conducted with 982 participants. Participants attended a mean of 2.2 tele-visits (SD 1.8; range 1–16 tele-visits; Table 2), and 2.5% of participants had seven or more tele-visits (Fig. 1). The mean (SD) length of time of an individual tele-visit was 17.6 min (SD 13.8; range 1.0–158.3 min; Table 2), and 14.2% of participants had an average visit time greater than 30 min (Table 3). The average total visit time per participant was 40.6 min with a range of 1.0 to 644.4 min (SD 51.2 min). Females spent more total time (44.3 min) in remote tele-visits compared with males (36.8 min, *p* = 0.02; Table 2).

**Table 2.** Remote Diabetes Care and Education Visits by Participant Characteristics

	Total number of tele-visits		Average length of tele-visits (in minutes)		Total time of tele-visits for each participant (in minutes)	
Totals						
*N* (minimum–maximum)	2203 (1–16)		(1.02–158.3)		(1.02–644.4)	
Mean (SD)	2.2 (1.8)		17.6 min (13.8)		40.6 min (51.2)	
Variable (*n*)	Mean	Unadjusted, *p*-value	Mean	Unadjusted, *p*-value	Mean	Unadjusted, *p*-value
Sex^a^						
Female (499)	2.3	NS	18.1	NS	44.3	0.02
Male (483)	2.1		17.1		36.8	
Type of diabetes^a^						
T1D (846)	2.1	<0.001	17.4	NS	36.6	<0.001
T2D (136)	3.4		19.2		65.3	
Insurance^b^						
Commercial (501)	2.0	^*^	16.4	^†^	32.4	^‡^
Medicare (286)	2.8	<0.001	21.1	<0.001	58.4	<0.001
Medicaid (195)	2.2		15.7		35.6	
Age (years)^b^						
18–35 (285)	1.8	§	14.8	^**^	26.2	^††^
36–54 (294)	2.2	<0.001	16.5	<0.001	37.4	<0.001
55+ (403)	2.6		20.4		53.1	

^*^ Commercial versus Medicaid, *p* = 0.33.

† Commercial versus Medicaid, *p* = 0.83.

‡ Commercial versus Medicaid, *p* = 0.73.

§Eighteen to 35 years old versus 36–54 years old, *p* = 0.02; 36–54 years old versus 55+ years old *p* = 0.04.

^**^ Eighteen to 35 years old versus 36–54 years old, *p* = 0.32.

†† Eighteen to 35 years old versus 36–54 years old, *p* = 0.02.

^a^ *t*-Test.

^b^ Analysis of variance.

NS, not significant.

**Table 3.** Mean Duration of Diabetes Care and Education Remote Visits

Average tele-visit time (in minutes)	Total number of tele-visits	Total number of participants, *n* (%)
<1.0	^a^	272 (21.7)
1.02–9.9	631	315 (32.1)
10.0–19.9	784	343 (34.9)
20.0–29.9	451	184 (18.7)
30.0–39.9	194	78 (7.9)
40.0–49.9	95	35 (3.6)
50.0–59.9	32	13 (1.3)
60.0–69.9	7	6 (0.6)
70.0+	9	8 (0.8)
Total	2203	1254

^a^ Could not be reached/did not receive education or support.

Adults with T2D comprised 13.8% of the participants. Those with T2D had a greater number of tele-visits compared with those with T1D (mean number of tele-visits: T2D = 3.4 vs. T1D = 2.1; *p* < 0.001) and a longer total duration of tele-visits (T2D = 65.3 min vs. T1D = 36.6 min; *p* < 0.001), respectively (Table 2). The discussions during the visits were categorized into topics shown in Table 4. A CDCES could discuss multiple topics with a participant within a single visit. The CGM education was discussed with 73.4% participants in a total of 1413 remote tele-visits. Preparation for provider telemedicine visits was the next most frequently discussed topic, with 58.7% of participants receiving instruction a total of 707 times.

**Table 4.** Frequency of Education Topics Discussed

Topics	No. of participants, *n* (%)	Frequency of topic discussed
CGM education	721 (73.4)	1413
Provider telemedicine visit preparation	576 (58.7)	707
Device download instruction provided	557 (56.7)	^a^
Pump education	488 (49.7)	841
Hypoglycemia	357 (36.4)	464
Lifestyle support	321 (32.7)	500
Management of glycemia	214 (21.8)	309
Medication management	196 (20.0)	346
Financial concerns: devices	143 (14.6)	160
Meter support	141 (14.4)	188
Psychosocial support	109 (11.1)	142
Sick day management	87 (9.9)	92
Insulin injection techniques	56 (5.7)	67
Appointment problems (re-schedule; cancellation)	45 (4.6)	45
Financial concerns: medications	48 (4.9)	53
Financial concerns: keeping appointments	11 (1.1)	11

^a^ Only counted once for each participant receiving instruction; some may have received instructions multiple times.

Device download instructions were provided to 557 participants (Table 4). Other topics included prevention and treatment of hypoglycemia, sick day management, medication management, insulin injection techniques, and lifestyle support (for the full list see Table 5).

**Table 5.** Description of Topics

Categorized as…	Tele-visit discussion included…
CGM education	Patient interest, benefits, features, training of new users, uploading, data review, troubleshooting
Provider telemedicine visit preparation	Use of video telemedicine platforms, appointment confirmation (date, time, telemedicine, or in-person), prescription requests, BG log collection, setup of EHR patient portal
Pump education	Patient interest, benefits, features, warranty, settings and upgrade options, uploading, data review, features, troubleshooting including but not limited to troubleshooting including but not limited to issues related to automated insulin delivery systems
Hypoglycemia	Awareness, prevention and treatment of low glucose, fear of hypoglycemia
Lifestyle support	Healthy eating, carbohydrate counting, gastroparesis, meal timing, physical activity
Management of glycemia	Natural progression of diabetes (including insulin resistance), T1D management, general education, glucose monitoring recommendations and strategies, glucose targets, factors affecting glucose/glucose variability
Medication management	How medications work, medication adjustments, side effects, insulin storage, insulin titration
Financial concerns: devices	Issues with insurance coverage of diabetes devices and supplies
Meter support	Troubleshooting meter (time and date corrections)
Psychosocial support	Distress related to COVID-19, diabetes distress, diabetes burnout, general distress, food insecurity
Sick day management	Glucose monitoring, ketone monitoring
Insulin injection techniques	Smart pens, how to inject insulin, injection sites, insulin pen use
Appointment problems (re-schedule; cancellation)	Needs to reschedule, wants to wait until in-person visit is possible, canceling appointment
Financial concerns: medications	Unable to afford medications
Financial concerns: keeping appointments	Unable to pay co-pay for appointment, felt telemedicine wasn't covered

BG, blood glucose; EHR, electronic health record.

### Differences by age

The mean total number of tele-visits was greater among participants with Medicare (2.8) compared with Medicaid and commercial insurance (2.2, *p* = 0.001 and 2.0, *p* < 0.001, respectively; Table 2). Those aged ≥55 years had a greater mean total number of tele-visits (2.6) compared with those aged 18–35 years (mean 1.8; *p* < 0.001) and those aged 36–54 years (mean 2.2; *p* = 0.041). The average duration of each visit in those aged ≥55 years was 20.4 min compared with those 18–35 years old (14.8 min), and those aged 36–54 years old (16.5 min), *p* < 0.001.

Older age was correlated with a greater number of tele-visits (*r* = 0.193; *p* < 0.001), higher average time for each remote visit (*r* = 0.204; *p* < 0.001), and greater total time spent in tele-visits (*r* = 0.263; *p* < 0.001). Participants of older age also had lower HbA1c values. The mean HbA1c for those aged 18–35 years was 8.8%, aged 36–54 years was 8.6%, and aged ≥55 years was 8.1% (≥55 vs. ≤54 years old, *p* < 0.001).

Older adults compared with younger adults were less likely to have access to a computer, tablet, smartphone, or internet (Table 6). Older age also correlated with a greater need for education and support related to CGM use, medication management, hyperglycemia, hypoglycemia, lifestyle support, and psychosocial support (Table 6).

**Table 6.** Topics Discussed with Remote Certified Diabetes Care and Education Specialists: Association with Older Age

Education topic	Correlation coefficients			
	Bivariate		Adjusted^a^	
	*r*	*p*	*r*	*p*
CGM education^*^	0.149	<0.001	0.105	0.001
How to take medications/medication management^*^	0.148	<0.002	0.078	0.015
Lack of computer/tablet/smart phone/internet access-connectivity^*^	0.13	<0.003	0.120	<0.001
Lifestyle support	0.115	<0.004	0.051	NS
Management of glycemia (hyperglycemia)^*^	0.079	0.01	0.063	0.048
Psychosocial support	0.066	0.04	0.027	NS
Hypoglycemia^*^	0.055	0.08	0.064	0.044
Financial concerns: devices	0.048	0.13	0.036	NS
Appointment preparation^*^	0.023	NS	0.061	0.058
Financial concerns: medications	0.015	NS	0.007	NS
Financial concerns: keeping providers' appointments	−0.003	NS	0.003	NS
Meter support	−0.004	NS	−0.015	NS
Sick day management	−0.012	NS	0.017	NS
Insulin injection techniques	−0.022	NS	−0.040	NS
Appointment problems (re-schedule; cancellation)	−0.037	NS	−0.029	NS
Pump education	−0.079	0.01	−0.003	NS

^*^ Partial/controlled correlation coefficients only displayed if significant at *p* < 0.06.

^a^ Adjusted for sex and diabetes type.

### Satisfaction survey results

In total, 211 surveys were collected and 198 were included for data analysis (response rate 35.5%). The mean age of responders was 52.7 years (SD 17.1); 78.0% had T1D, 56.1% were female, and 44.9% had commercial insurance. Participants had a mean HbA1c of 8.0% (SD 1.6), 64.1% used CGM, and 49.5% used an insulin pump. Participants who completed the survey had a mean of 2.7 (SD 2.2) tele-visits; the average visit was 19.5 min (SD 13.5 min), and the total average time was 54.3 min (SD 71.9 min). Surveys were excluded if: (1) the participant did not recall having a CDCES visit, (2) the survey was not fully completed, (3) the survey was a duplicate, or (4) the participant had a diagnosis other than T1D or T2D.

Although the majority of participants stated they were satisfied with the remote education visit (93.4%), those who were younger had greater satisfaction (*r* = 0.175; *p* = 0.014). Younger participants felt that the remote education visits were convenient (*r* = −0.167, *p* = 0.019) and that the remote visits saved them time (*r* = −0.244, *p* = 0.001; Table 7). Those with Medicaid were more likely to agree or strongly agree with the statement “In the past, cost was a barrier to receiving diabetes education” compared with those with private insurance (*p* = 0.005). Importantly, 86.9% of participants agreed with the statement “my concerns were addressed” during the remote visit and 81.3% agreed that the remote visit satisfied their diabetes education needs.

**Table 7.** Correlation of Patient Satisfaction Survey Items with Age

Item	Total % agreed	Total mean score	r (*p*)
I would participate in remote education again	84.3	4.07	−0.263 (<0.001)
The remote visit satisfied my diabetes education needs	81.3	3.94	−0.260 (<0.001)
Would recommend remote diabetes education to a friend or family member	76.8	3.93	−0.257 (<0.001)
Remote education visit saved me time	89.4	4.18	−0.244 (0.001)
I felt comfortable using telehealth	82.3	3.89	−0.234 (0.001)
Used language I could understand	97.0	4.44	−0.225 (0.001)
Lack of physical contact was not a problem	84.3	4.05	−0.218 (0.002)
In general, satisfied with the remote education visit	93.4	4.28	−0.175 (0.014)
Remote education visit was convenient	91.4	4.26	−0.167 (0.019)
As satisfying as in-person visits	61.6	3.52	−0.152 (0.033)
Could see clearly	28.8	1.45	−0.138 (0.052)
Could hear clearly	92.9	4.37	−0.136 (0.057)
My concerns were addressed	86.9	4.12	−0.134 (NS)
Helped me better prepare for the provider visit	73.7	3.67	−0.102 (NS)
I was satisfied with my in-person education before this visit	77.3	3.58	−0.088 (NS)
In the past, cost was a barrier to receiving education	19.7	2.20	−0.009 (NS)
Helped me better manage my diabetes	63.1	3.60	−0.002 (NS)

## Discussion

Early in the pandemic, over 5 months, 78.3% of referred patients completed remote CDCES visits and most had two or more visits based on participant need for education and support. Older adults had more remote CDCES visits, the tele-visits on average lasted longer, and the total time with a CDCES was longer than in younger adults. Overall, 85.7% of the individual remote visits lasted <30 min. Importantly, almost none of these CDCES tele-visits would have been billable to insurance since most insurers will only reimburse for education sessions that last for at least 30 min. However, most participants had multiple tele-visits, which in total lasted >30 min.

Deficits in cognitive function are common in aging, especially in the presence of diabetes, and include impaired sustained and selective attention and working memory.^29–32^ Clinically significant cognitive impairment was found in 48% of a sample of individuals with diabetes ≥60 years old.^30^ More frequent, shorter sessions may be more productive and provide more opportunities for reinforcement than longer sessions in older adults since attention tends to fade and information overload is more common with longer remote education visits.

Frequent but shorter tele-visits are easier to schedule and attend than frequent in-person visits, given transportation, staffing, and other operational and social considerations. This experience suggests that Medicare and other insurers should re-evaluate their policies that preclude reimbursement for individual DSMES visits with a duration of <30 min. We believe that shorter, more focused remote discussions can improve comprehension and self-management, but this was not tested. Studies evaluating the efficacy of shorter and longer DSMES visits are needed in older adults, but they are difficult to perform in real-world settings.

Remote CDCES visits have been used successfully to initiate and train adults in the use of CGM.^33^ The CGM has been shown to reduce hypoglycemia in older adults with both T1D and insulin-treated T2D and increases the sense of safety for the PWD.^34–37^ Since initiating and sustaining CGM use in people with T1D is a priority, it was requested that the remote CDCESs discuss CGM with all individuals with T1D not using CGM. It is, therefore, not surprising that CGM use was a frequent topic. We have previously shown that older adults with T1D have more barriers to starting CGM,^38^ many of which can be addressed with more education and support.

We were not able to assess whether individuals started and sustained CGM use as a result of a CDCES remote visit since providers were also recommending CGM and, simultaneously, there were other policy changes that made CGM more available and affordable for many older adults. However, anecdotally, participants told providers that their sessions with the CDCESs were helpful in better understanding and using CGM.

The needs of PWD vary over time due to changes in life circumstances, the development of complications, and the availability of new technologies and medications that require education and training for proper use and optimal benefit. The Consensus report of the American Diabetes Association, the Association of Diabetes Care and Education Specialists, the Academy of Nutrition and Dietetics, the American Academy of Family Physicians, the American Academy of Physician Assistants, the American Association of Nurse Practitioners, and the American Pharmacists Association recommend DSMES services at initial diagnosis, annually, when new complicating factors affect self-management and during transitions.^39^

Medicare covers 10 h of DSMES in the 1st year that beneficiaries receive this service and 2 h/year for each year thereafter.^40^ Although individuals usually require more education initially, there are times in subsequent years, such as when new diabetes technologies are prescribed or when complications develop, that >2 h of education per/year are needed. This is especially true for older adults who are experiencing cognitive decline, when more education is needed for both the PWD and their caregivers to ensure safety.

In the short time that many older adults interacted with the remote CDCESs, it was found that they needed more instruction related to technology when compared with those in younger age groups. Data are not available spanning a full year, but it is likely that 2 h of education time is insufficient for many older adults, especially if starting CGM or a new insulin delivery system.

All staff have the necessary technology to complete remote DSMES visits (computer with secure remote video capability). Although some PWD only have telephone access, increasingly they are obtaining access to a smartphone, tablet, or computer technology. Since our results suggest that PWD benefit from remote telephone and/or video DSMES visits as well as in-person visits, both should be equally reimbursed.

Adults with T2D had more DSMES tele-visits and longer total time was spent with the CDCESs. This is likely due to the fact that individuals with T2D were referred by their medical providers, because they identified specific education needs. In contrast, all adults with T1D and upcoming provider visits were referred without consultation with their providers. It is hypothesized that, if a selection process was used for referral of those with T1D, the total duration of tele-visits would also be longer.

Lower participant satisfaction with remote diabetes education in older adults was observed. Reasons for this may include less knowledge and comfort with using devices than those who are younger. It is possible that, over time and with more experience and use of technology, this will change. Other contributing factors were lesser availability of computers and video equipment in the home, concern for privacy, and visual and/or hearing impairments.

This report has several limitations. Remote CDCESs were only available for a limited period of time (5 months). These data are from a single center, glycemia data and quality of life data were not collected, and potential long-term benefits could not be measured. A “control” (in-person) group was not possible since the use of the remote CDCESs was triggered by the start of the COVID-19 pandemic and the inability to conduct routine in-person visits.

These data are mostly from adults with T1D or T2D on complex insulin regimens, so these results cannot be generalized to others with T2D. The remote DSMES was provided without charge so that all adults could participate without incurring a cost, acknowledging that some would not have participated if there had been a charge. The relatively small number of participants who completed the satisfaction survey was also a limitation.

There are also strengths of this article. Importantly, it is the first real-world study, to our knowledge, involving a large number of PWD (*n* = 1254), to present data related to the length of time needed for remote CDCES visits, and included PWD over the adult lifespan.

## Conclusions

Older adults with T1D or T2D on complex insulin regimens needed a greater number and duration of remote visits. This population may do better with more frequent, shorter tele-visits. Since the majority of the remote visits lasted <30 min, these services would not have been reimbursed under current insurance policies. Changes in reimbursement for needed remote education and support services should be considered. Reimbursement should be based on cumulative time rather than by time spent in an individual visit.

The level of satisfaction was high with diabetes education tele-visits, supporting this method of intervention. However, although there are many benefits associated with the use of remote visits, there are disparities in the ability to access these services. Further studies are needed to examine the adaptations and approaches that could improve the use of telehealth for older adults.

## Abbreviations Used

CDCES: Certified Diabetes Care and Education Specialist
CGM: continuous glucose monitor
DSMES: Diabetes self-management education and support
HbA1c: glycosylated hemoglobin
PWD: people with diabetes
SD: standard deviation
T1D: type 1 diabetes
T2D: type 2 diabetes
